# Remarks on Suprapubic Lithotomy*Abstract of a lecture delivered at the Chicago College of Physicians and Surgeons. (The author’s views of Suprapubic Lithotomy in general have been presented in his recent text-book on “Genito-Urinary and Sexual Diseases.” F. A. Davis Co.)

**Published:** 1899-12

**Authors:** G. Frank Lydston

**Affiliations:** Professor of the Surgical Diseases of the Genito-Urinary Organs and Sypilology in the Medical Department of the State University of Illinois; 110 State Street, Chicago


For Texas Medical Journal.

### Remarks on Suprapubic Lithotomy.*

BY G. FRANK LYDSTON, M. D.,
Professor of the Surgical Diseases of the Genito-Urinary Organs and Sypilology in the Medical Department of the State University of Illinois.

The high operation for stone illustrated the mutability of surgical fashion. Introduced about the middle of the sixteenth century by Pierre Franco, it fell into disuse and remained traditional until quite recently. It was revived, mainly through the efforts of Petersen, whose systematic technique has made the method quite popular. Considering the progress of abdominal surgery, it is not surprising that the simplest and most direct operation for vessical calculus should become a recognized and reliable operation. The objections formerly advanced against it do not now hold good. Modern antisepsis and asepsis have modified our notions of suprapubic lithotomy as decidedly as other operations.

The only unfortunate circumstance in the revival of the suprapubic operation is the fact that its neglect in past years has been latterly replaced by over-enthusiasm. So reckless has this become that some surgeons propose the high operation as a matter of routine, irrespective of the size of the stone or the patient's condition. Considering the small mortality of litholopaxy and lateral lithotomy in young subjects, it is hardly fair, as yet, to ask that we universally substitute for them, suprapubic lithotomy. It is noteworthy that in children the mortality has been twelve per cent., which record will doubtless be improved upon.

In some severe cases suprapubic section, if properly performed, affords the best chance of recovery. Cases of serious kidney complication where operation is demanded, very large stones with severe cystitis, tumors with or without stone, foreign bodies in the bladder,

---

*Abstract of a lecture delivered at the Chicago College of Physicians and Surgeons. (The author's views of Suprapubic Lithotomy in general have been presented in his recent text-book on "Genito-Urinary and Sexual Diseases." F. A. Davis Co.)

and ensacculated stones are best suited to the suprapubic operation in both sexes. With many surgeons the favorite operation for foreign bodies will, however, probably always be the median section, on account of its simplicity and safety. Very large stones, *i. e.*, those of a diameter of one inch and a half upward, stones too hard for crushing, and stones with known uncrushable nuclei, or nuclei that cannot safely be crushed—should usually be operated by the high method. Stones may be removed in this way where the lateral operation would probably prove fatal. The danger of lateral lithotomy increases rapidly with the size of the stone, which is not so true of the suprapubic operation, although on account of associated conditions the prospect is not usually so good in large as in small stones. The inteference with the urethra, prostate, and vesical neck involved in lateral section, is more likely to induce urine fever than suprapubic section, and this is especially true where there is decided secondary involvement of the kidneys. This is explained by the extreme sensitiveness and close association of these parts with the sympathetic system, as compared with the structures involved in the high operation.

*Operation.*—Petersen's operation is performed as follows: The patient is prepared as for ordinary lithotomy. After thorough scrubbing, the abdomen is cleansed with a weak bichloride or strong carbolic solution, as in preparation for celiotomy, and the hair shaved off the pubes. The patient is placed upon his back. The bladder is now washed out with a warm antiseptic solution (ac. carbol., ac. boric., borax, or salicylate of soda) via a soft catheter.

Petersen's rectal bag (colpeurynter) is now introduced and filled with ten or twelve ounces of water, thus pushing the bladder forward and upward. Care is necessary in this procedure; the injection of an excess of water or too rapid filling of the bag may rupture the rectum—this has happened to several competent operators. The bladder is next filled with from eight to twelve ounces of a mild antiseptic solution, thus bringing the viscus upward and forward still more prominently. Caution is also necessary at this point, lest the bladder be ruptured.

A median incision is now made beginning about three to three and one-half inches above, and terminating at the symphysis, involving the skin and cellular tissues down to the linea alba. The deep incision should now be made; if practicable, exactly between the recti muscles. There should be as little tearing and bruising of the tissues as possible. After the muscles have been divided the transversalis fascia is exposed and should be carefully incised upon a director, beginning at the lower angle of the wound, at which point it is usually impossible to pick up the peritoneum. Beneath this fascia in the prevesical space—the cavity of Retzius—is a quantity of fat that should be pushed or rolled upward out of the way,—*enroulement*,—as practiced by Guyon. Should the peritoneum be accidentally cut, the wound is immediately sewed with fine catgut and the operation proceeded with.

The bladder is now brought plainly into view. Curved needles threaded with heavy silk are next passed through the bladder walls, one upon each side and parallel with the line of incision, and brought out so as to leave a long double thread; the ends of these threads are tied so that the bladder may be held against the edges of the wound by the loops in the hands of assistants. A free vertical incision is next made into the bladder and its interior thoroughly examined, both by the eye and finger. Should the parts be seen with difficulty, the Trendelenburg position will draw the bladder into plainer view by the suction-force of the receding abdominal viscera. The stone having been removed by fingers or forceps, the bladder is once more washed out, a catheter introduced, and the bladder sutured, care being taken to suture through the muscular walls only, and not through the mucous membrane. A small quantity of gauze is left just above the pubis to drain the prevesical space, the external wound being sutured as in ordinary celiotomy.

It is recommended by some that a suprapubic drainage tube be left in the bladder; but this is unnecessary where the bladder is sutured. Whenever the vesical wound is closed, a drainage tube should usually be inserted through a boutonniere in the perineum, though a soft catheter per urethram may suffice.

Experience has led the author to modify the Petersen method somewhat. The rectal bag is unnecessary and dangerous. The bladder is always accessible unless contracted and hardened, under which circumstances the rectal bag is of little use anyway. The fingers of an assistant in the rectum fulfill the indications much better than the rectal bag. With the Guyon method of enroulement, the peritoneum is rarely seen. Should it be cut, it should be stitched and the wound packed for two or three days before entering the bladder, if the latter be septic. This operation in two stages—first employed in France—should be the operation of election in feeble patients with septic bladders. The danger of infection is made practically nil by it. Where the section *a deux temps* is deliberately chosen the bladder should be exposed over as wide an area as possible, and the packing allowed to remain for five days. In very large stones, in tumors, and where the peritoneum is low down or the bladder contracted, the author has practiced the following method: The bladder is exposed in the usual manner, and the peritoneum deliberately incised for as great an extent as deemed necessary. The bladder is drawn out of the incision to the required extent and the peritoneum stitched around it, lateral temporary retention sutures being placed in the bladder and edges of the abdominal wound. The bladder is packed about with iodoform gauze and the usual dressings applied. On the fifth day the operation may be proceeded with. There is now plenty of room, a large surface of the bladder being uncovered by peritoneum. Stitching the bladder is thus made a very simple matter. In the ordinary operation *a deux temps* both the preliminary and secondary incisions may be done under Schleich's method, thus obviating the dangers of general anesthesia. As already noted, the Trendelenburg posture greatly facilitates all operations upon the prostate. In extremely septic bladders through-and-through drainage is best. The author does not advocate vesical suture save in exceptional cases where infection is not to be apprehended. Where suture is performed, the wound leading down to the line of suture should be carefully packed, secondary silkworm-gut sutures being utilized for the closure of the external wound as soon as it is deemed safe to do so; *i. e.*, in four or five days. Even then it is wise to leave a bit of gauze drainage in the lower angle of the wound, leading down to the cavity of Retzius.

Observations made during experiments on intestinal suture in 1881, showing the ready repair of accidental wounds of the bladder, led Rydygier of Krakow, to the idea that the tendency to adhesion of the peritoneum could be utilized in cystotomy. To prove this he made a series of experiments on dogs, and encouraged by the results, operated on a boy thirteen years of age. Rydygier's method of operating is, briefly, as follows:

The patient is prepared as for laparotomy, a catheter is passed into the bladder, and the latter is washed out with a warm solution of boric or salicylic acid, and then partly filled with the same solution. A section is made on the linea alba, as usual. After letting out the solution by means of a catheter, the bladder is drawn out by a silk noose, and iodoform gauze is solidly packed in between the bladder and the edge of the wound. An incision one and a half inches long is made in the bladder, and the finger is inserted and along it the forceps. The bladder is again washed out by the catheter with the above solution, which may overflow the wound of the bladder without entering the cavity of the abdomen. Rydygier recommends applying the furrier's suture according to Czerny's method. This suture has this advantage: that the more the bladder distends, the more the suture tightens. It is necessary to keep the catheter in the bladder for eight to twelve days. Rydygier's method can particularly be applied to the entire removal of tumors of the bladder, and it may also be of use in wounds of this viscus.

The after treatment of suprapubic cystotomy consists of ordinary antiseptic dressings, changed as required, siphon-drainage with daily vesical irrigation, and removal and cleansing of the catheter or drainage tube. At the end of a week or ten days it is usually safe to allow the perineal tube or catheter to remain out for forty-eight hours. If no urine escapes through the abdominal wound at the end of that time, the drainage tube may be dispensed with permanently. The patient should be instructed to urinate at short intervals for a few weeks, to prevent undue tension upon the vesical cicatrix. The bladder should be washed out daily with a weak borated or carbolized solution for some time.

In occasional cases either a truss or a radical operation will be necessary later on account of hernia from yielding of the cicatrix.

In occasional cases removal of large stones suprapubically is followed by a fatal result, because of certain conditions in which the presence of the stone is apparently necessary to the preservation of the integrity of the vesical wall. The pressure of the stone, while disastrous, apparently prevents sloughing of the portion of the bladder on which it rests. The following case would appear to be an illustration of this:

Case.—X., a man 65 years of age, consulted the author regarding vesical irritation of nearly ten years' duration. Exploration revealed stone of large size, whether one or more could not be determined, because of the large size of the calculous mass and the contracted state of the bladder. Suprapubic section was performed and two large stones weighing 1500 grains removed with some difficulty, but without injury to the incised portion of the vesical wall, save that incidental to the incision itself. A lithotomy-scoop and the fingers only were used in extracting the calculi. The bladder was adherent to the edges of the stone where they came in apposition. The peritoneum was not seen during the operation. The patient died of septic peritonitis on the fourth day after operation. On the second day the vesico-rectal septum sloughed, and enemata passed freely from the rectum into the bladder. No autopsy could be secured.

In the foregoing case sloughing of the vesical wall was probably due to the sudden removal of the pressure of the calculi and incidental acute sepsis, the phenomena being similar to those occurring after the sudden evacuation of retained urine in old septic bladders. The peritonitis was due, in the author's opinion, to slight laceration of the degenerated and friable vesical wall, with its peritoneal investment, by the tearing away of the adherent calculi. Adhesion of calculi to the vesical walls is certainly a very rare condition, but one that undoubtedly existed in this case, as shown both by the conditions found during removal and the appearance of the calculi after removal. The case is a very instructive one and would naturally lead to the suspicion that quite a proportion of the by no means rare cases of fatal peritonitis following stone operation may not be due to similar conditions.

110 State Street, Chicago.

